# Long-term impact of Diabetes Prevention Program interventions on walking endurance

**DOI:** 10.3389/fpubh.2024.1470035

**Published:** 2024-12-18

**Authors:** Medha N. Munshi, Elizabeth M. Venditti, Ashley H. Tjaden, William C. Knowler, Edward J. Boyko, Roeland J. W. Middelbeek, José A. Luchsinger, Christine G. Lee, Helen P. Hazuda, Marcel E. Salive, Sharon L. Edelstein, Thomas W. Storer

**Affiliations:** ^1^Joslin Diabetes Center, Beth Israel Deaconess Medical Center, Harvard Medical School, Boston, MA, United States; ^2^Department of Psychiatry, University of Pittsburgh School of Medicine, Pittsburgh, PA, United States; ^3^DPP/DPPOS Coordinating Center, The Biostatistics Center, Milken Institute School of Public Health, The George Washington University, Rockville, MD, United States; ^4^DPP/DPPOS Coordinating Center, Biostatistics Center (Consultant), The George Washington University, Rockville, MD, United States; ^5^Seattle Epidemiologic Research and Information Center, VA Puget Sound, Seattle, WA, United States; ^6^Departments of Medicine and Epidemiology, Columbia University Irving Medical Center, New York, NY, United States; ^7^National Institute of Diabetes and Digestive and Kidney Diseases, NIH, Bethesda, MD, United States; ^8^The University of Texas Health Science Center at San Antonio, San Antonio, TX, United States; ^9^Division of Geriatrics and Clinical Gerontology, National Institute on Aging, Bethesda, MD, United States; ^10^Brigham and Women’s Hospital, Harvard Medical School, Boston, MA, United States

**Keywords:** diabetes, walking endurance, aging, six-minute walk test, lifestyle

## Abstract

**Objectives:**

Type 2 diabetes (T2D) and prediabetes are associated with poor walking endurance, a marker of physical function. We aimed to examine the long-term effects of metformin or intensive lifestyle intervention in adults at high risk of T2D on their 6-min walk test (6MWT) performance.

**Methods:**

Participants were randomized in the 3-year Diabetes Prevention Program (DPP) to one of the three groups: lifestyle intervention, metformin, or placebo, and were subsequently followed in the DPP Outcomes Study. A 6MWT was conducted 20 years after randomization. Associations between DPP interventions and 6MWT completion (achieving a distance ≥200 m) were assessed using logistic regression. Among the test completers, differences in distance walked (6MWD) were evaluated using multivariable linear regression. Additional variables of interest included concomitant measures of body mass index (BMI) and grip strength along with mean measures of HbA1c and self-reported physical activity (PA).

**Results:**

Data on 1830 participants were analyzed. The interventions were not associated with test completion or the 6MWD among test completers (362, 364, and 360 m in the lifestyle, metformin, and placebo groups, respectively, *p* = 0.8). Age, education, grip strength, and PA were each significantly associated with the 6MWT completion and the 6MWD after adjustment. Grip strength, PA, and education were positively associated with the 6MWD, while age, BMI, and HbA1c were negatively associated with the 6MWD.

**Conclusion:**

We confirmed that the 6MWT is related to other measures of physical ability such as PA and grip strength in persons at risk for and with T2D, suggesting potential long-term benefits of maintaining a healthy lifestyle. However, we did not observe a sustained effect of the original randomized interventions.

**Clinical trial registration:**

http://www.clinicaltrials.gov/ct/show/NCT00004992, identifier DPP NCT00004992; http://www.clinicaltrials.gov/ct/show/NCT00038727, identifier DPPOS NCT00038727

## Introduction

Both prediabetes and type 2 diabetes mellitus (T2D) are independently associated with reduced physical activity and a higher risk of disability (1–9). Physical fitness, including cardiopulmonary endurance and skeletal muscle strength and power, are important health-related attributes for mitigating disability in older people. Disability in the form of mobility limitation is associated with functional decline (10, 11) and loss of independence among older adults (10, 12). Assessed by walking speed, impaired mobility is well associated with disability in activities of daily living (11, 12), cardiovascular disease, and mortality (13, 14). Mobility, in the context of physical activity, is well-known as a positive determinant of mental health that is associated with psychological (15, 16) and social wellbeing (17, 18).

The 6-min walk test (6MWT) measures how far a person can walk in 6 min under standardized conditions. Since the test is submaximal, it may better indicate an individual’s ability to perform activities of daily living than maximal exercise testing (19). The test is clinically validated to objectively evaluate endurance in older adults (20). The total distance walked in the 6MWT (6MWD) diminishes with greater age, poor overall health status, T2D, a low score on cognitive function tests, and a high C-reactive protein concentration. The 6MWT is also associated with all-cause mortality (21–24). The majority of studies evaluating 6MWT performance are conducted on individuals with heart, lung, or neuromuscular diseases, who are often older adults. Although T2D is a known risk factor for poorer performance on the 6MWT, the effects of diabetes prevention interventions on the 6MWT performance among adults at risk for diabetes are not well-known.

The effect of metformin use on physical functioning has shown mixed results, with benefits shown on some parameters of exercise capacity, including VO_2peak_, which is associated with 6MWT performance (25–27). An epidemiological study in women with T2D suggested a beneficial effect of insulin sensitizers, particularly metformin, on preventing the loss in gait speed seen with aging (28). It is unclear whether longer-term metformin use affects physical endurance as measured by the 6MWT. Moreover, these previous studies examined individuals with T2D; thus, it is not known how metformin or multicomponent lifestyle interventions aimed at improving eating habits, increasing physical activity, and producing modest weight loss over time, may affect longer-term physical performance in individuals at high risk of T2D.

The Diabetes Prevention Program Outcomes Study (DPPOS) is a long-term follow-up of the Diabetes Prevention Program (DPP). The DPP evaluated the role of metformin and an intensive lifestyle intervention (ILS) in preventing or delaying the onset of T2D in high-risk adults. Data from longer follow-ups in DPPOS showed a sustained impact of interventions administered during the DPP on frailty (29). The goal of this analysis was to determine the effects of treatment with metformin or ILS, compared to placebo, on walking endurance as measured using the 6MWT as part of the long-term follow-up in DPPOS participants. Understanding the effects of metformin therapy and ILS on walk performance may help to prevent frailty and loss of mobility in older adults with or at risk for T2D.

## Methods

### DPP/DPPOS randomized interventions

The DPP (July 1996 to July 2001) was a 27-center randomized controlled trial that compared the efficacy of metformin or ILS with placebo to prevent or delay T2D among 3,234 adults aged ≥25 years old who were overweight or obese and had prediabetes at baseline (30–32). ILS participants were offered an individualized 16-lesson curriculum over 24 weeks followed by monthly sessions through the DPP. The curriculum focused on diet, exercise, and behavior change, guiding participants to follow a low-fat, low-calorie diet (<25% kcal from fat) and perform ≥150 min/week of physical activity, with the primary goal to achieve ≥7% weight loss from baseline weight (33). Metformin participants were assigned to take blinded 850 g metformin twice daily; placebo participants were assigned a matching placebo pill twice daily. Both the metformin and placebo groups received written standard lifestyle recommendations and a one-on-one lifestyle session annually. After the DPP ended, the metformin and placebo groups were unmasked, and all participants were offered a modified lifestyle program for 6 months. Subsequently, 2,779 participants consented to participate in the DPP Outcomes Study (DPPOS), and all participants were offered quarterly lifestyle classes. Open-label metformin was continued for those originally randomized to metformin, placebo was discontinued, and ILS was provided as semi-annual group-based classes (34).

The study protocols for DPP/DPPOS are publicly available at https://dppos.bsc.gwu.edu/web/dppos/dpp.

### Study participants

This analysis was limited to those DPP/DPPOS participants who completed a 6MWT eligibility assessment at their DPPOS Year 15 (calendar years 2016–2017) follow-up visit (19–21 years after randomization).

### Procedures 6MWT

#### Assessor training

Study coordinators and lead examiners at each DPPOS site completed centralized in-person training and practice in 6MWT administration until proficiency was achieved. One of the authors (TWS) with extensive 6MWT expertise served as the master trainer (35). All training materials were developed from published, standardized guidelines (19). A performance checklist (Supplementary Material) was developed to ensure the ability of 6MWT assessors to proficiently administer the test and to train new examiners at individual study sites.

#### Standardized walking course and equipment

Twenty of the twenty-four sites that participated in the 6MWT examination used a course length of 20 m; the remaining sites used 10-m courses because of space constraints. Turn-around zones of 2.5 m at each end were provided on all courses. The courses were measured either with a non-distensible tape measure or a measuring wheel; a hand-held stopwatch was used to monitor and record the actual time walked. Clinic staff were instructed to administer the test indoors along a long, flat, straight, enclosed corridor with a hard surface that is seldom traveled. In one clinic with a year-round temperate climate, the 6MWT was administered outdoors.

#### Administering the 6MWT

The 6MWT was first assessed at the DPPOS Year 15 follow-up visit (19–21 years after randomization). Participants rested in a seated position for 5 min before the start of the test. Heart rate and blood pressure were measured along with a brief assessment of contraindications. Participants were not permitted to warm up and only one test was given. Test administrators demonstrated one lap of the walk test and used the same standardized script to instruct participants in the performance of the test (19). Participants were asked to “…walk as *far as possible at a speed that you can maintain safely for 6 min.”* Lengths were recorded on a score sheet throughout the test. Participants were allowed to use usual walking aids, such as canes or walkers, and stop and rest as necessary, and allowed to discontinue the test at any time. Standardized words of encouragement were given at the end of 1 to 5 min, and again at 5 min 45 s (19). The stopwatch was stopped at approximately 6 min, and a marker was placed at the toe of the foot striking the floor on the command stop. Participants were returned to a seated position for assessment of heart rate, RPE, and signs and symptoms. Participants completed the Borg 0–10 scale for rating of perceived exertion (RPE) after the test (36, 37).

#### Distance walked (6MWD)

Completed lengths were recorded and multiplied by the course length. Distance walked beyond the last complete length was added to the distance calculated from completed lengths, representing the distance walked in 6 min (6MWD). For participants with stopwatch times that exceeded 6 min, the total distance measured was interpolated to exactly 6 min. Participants with stopwatch times exceeding 7 min were excluded from analyses assessing the 6MWD.

### Other measures

Physical activity data were collected via the Modifiable Activity Questionnaire (MAQ) annually and the median of all available responses [median = 17; range = 4–19 observations per participant] between the DPP baseline and DPPOS Year 12 (3 years prior to the 6MWT) of metabolic equivalent (MET)-hours of leisure activity was used as a measure of habitual activity level (38–40). Diabetes at the concurrent visit was ascertained using ADA criteria as previously described (30, 41). A composite variable for neuropathy signs and symptoms was defined by signs present based on a monofilament test at the concurrent visit or symptoms from Michigan Neuropathy Screening Instrument (MNSI) at the DPPOS Year 13 visit (2 years prior to 6MWT). Participants self-reported recent (within the past 12 months) hospitalizations and falls, as well as ever having a hip replacement. Stroke, CHF (congestive heart failure), MI (myocardial infarction) was adjudicated by review of medical records by reviewers masked to treatment assignment. A composite health issue variable was created to combine recent hospitalization, history of hip replacement, history of stroke, CHF or MI, and recent falls. The FEV1/FVC ratio was collected using a spirometry test at the concurrent visit. Grip strength was assessed with a handheld dynamometer using standardized procedures at the concurrent visit. Total metformin exposure was defined as the number of years taking study or non-study metformin. Cumulative glycemic exposure is defined as the mean HbA1c for all available semi-annual and annual visits between the DPP baseline and the time of 6MWT assessment (median = 24, range = 9–39 visits).

### Statistical analysis

Descriptive statistics were used to examine the characteristics of the study population at baseline and at the time of their DPPOS Year 15 follow-up visit, which occurred 19–21 years after randomization. The baseline characteristics in the analytic cohort were also compared to participants from the original randomized DPP cohort who did not complete the 6MWT. Comparisons between groups were computed using ANOVA for continuous variables and chi-squared tests for categorical variables. Participants with the shortest distance walked (<200 m) were categorized as poor performers (approximately the 10th percentile test completers). Among all participants in the analytic cohort, logistic regression models adjusted for randomization group, age, sex, and ethnicity were used to examine the association of select characteristics at baseline and at follow-up with the ability to complete the 6MWT (completion) with a distance of ≥200 m. Among all participants who completed the test (regardless of distance walked), the mean distance walked (m) was compared by participant characteristics.

General linear regression models were used to examine the differences in total 6MWD by randomization group, diabetes, cumulative glycemic exposure, cumulative metformin exposure, and concurrent BMI. Several possible confounders were identified *a priori* for the base multivariable linear regression model (Model 1) for the base multivariable linear regression model. Subsequent models included additional covariates identified in a stepwise manner as possible confounders if they were significantly associated with distance walked after adjustment for randomized assignment. Model 2 adds education and health-related variables including smoking, concurrent BMI, height, and waist circumference. Finally, Model 3 adds concurrent grip strength, habitual physical activity, the presence of neuropathy signs/symptoms at the time of assessment, the presence of a health issue, and cumulative glycemic exposure. Effect modification by age, sex, ethnicity, and diabetes was tested.

## Results

Of the 3,234 participants randomized in the DPP from 1996 to 1999, 2,130 completed their DPPOS Year 15 annual visit between July 2016 and November 2017 when the 6MWT was conducted. The analytic group assessed for 6MWT eligibility represents 1,830 (86%) of the 2,130 individuals who completed a DPPOS Year 15 visit (Supplementary Figure 1). Of these 1,830 participants, 1,697 (93%) completed the 6MWT. Three participants had stopwatch times that exceeded 7 min and were excluded from subsequent analyses. Thus, 1,830 participants were included in analyses with test completion as the outcome, and 1,694 participants were included in analyses with total distance walked in 6 min as the outcome. Supplementary Table 1 compares the baseline characteristics of participants included in these analyses with those not included among the originally randomized DPP cohort.

Participant characteristics at the DPP baseline and at the DPPOS Year 15 visit are shown in Table 1 for 1,830 participants. Percent and mean values of the majority of variables were similar among the three randomization groups at baseline. The only nominally significant (*p* < 0.05) difference among the randomization groups was for height, which was highest in the metformin group.

**Table 1:** Participant characteristics at DPPOS year 15 by treatment assignment group among all participants with a visit (*N* = 1830).

	All (*N* = 1830)	Lifestyle (*N* = 588)	Metformin (*N* = 617)	Placebo (*N* = 625)	*p*-value
*Baseline characteristics*					
Age (years)	49.6 ± 9.2	49.4 ± 9.7	50.1 ± 8.9	49.2 ± 9.0	0.277
Age group					0.143
25–44 years	559(30.5%)	196(33.3%)	178(28.8%)	185(29.6%)	
45–59 years	1,015(55.5%)	301(51.2%)	353(57.2%)	361(57.8%)	
60+ years	256(14.0%)	91(15.5%)	86(13.9%)	79(12.6%)	
Sex (Female)	1,261(68.9%)	408(69.4%)	412(66.8%)	441(70.6%)	0.338
Ethnicity					0.344
Non-Hispanic White	957(52.3%)	294(50.0%)	331(53.6%)	332(53.1%)	
Non-Hispanic Black	338(18.5%)	107(18.2%)	123(19.9%)	108(17.3%)	
Hispanic	308(16.8%)	101(17.2%)	100(16.2%)	107(17.1%)	
American Indian	134(7.3%)	46(7.8%)	40(6.5%)	48(7.7%)	
Asian	93(5.1%)	40(6.8%)	23(3.7%)	30(4.8%)	
Education (College or more)	1,377(75.2%)	441(75.0%)	476(77.1%)	460(73.6%)	0.345
*Year 15 characteristics*					
Age (years)	68.6 ± 9.2	68.5 ± 9.6	69.1 ± 8.8	68.3 ± 9.0	0.269
Smoking					0.169
Never	1,112(60.8%)	352(59.9%)	384(62.2%)	376(60.2%)	
Past	663(36.2%)	221(37.6%)	220(35.7%)	222(35.5%)	
Current	55(3.0%)	15(2.6%)	13(2.1%)	27(4.3%)	
Height (cm)	**164.1 ± 9.1**	**164.0 ± 8.9**	**164.8 ± 9.3**	**163.5 ± 9.2**	**0.046**
BMI kg/m^2^	32.3 ± 6.7	32.1 ± 6.7	32.0 ± 6.6	32.8 ± 6.8	0.061
BMI categories					0.150
<30 kg/m^2^	765(41.8%)	258(43.9%)	274(44.5%)	233(37.3%)	
30–35 kg/m^2^	510(27.9%)	159(27.0%)	162(26.3%)	189(30.2%)	
35–40 kg/m^2^	322(17.6%)	105(17.9%)	104(16.9%)	113(18.1%)	
40+ kg/m^2^	232(12.7%)	66(11.2%)	76(12.3%)	90(14.4%)	
Waist (cm)	106.5 ± 14.2	106.2 ± 14.4	106.0 ± 13.6	107.3 ± 14.6	0.206
Fasting glucose (mg/dl)	**126.0 ± 38.1**	**128.8 ± 40.4**	**119.5 ± 30.6**	**129.7 ± 41.7**	**<0.001**
Confirmed diabetes	**1,178(64.4%)**	**369(62.8%)**	**379(61.5%)**	**430(68.8%)**	**0.017**
Neuropathy signs/symptoms* (Present)	848(47.8%)	276(48.2%)	288(48.2%)	284(47.2%)	0.925
Recent hospitalizations (Yes)	65(3.6%)	19(3.2%)	22(3.6%)	24(3.8%)	0.846
Fall in past 12 months (Yes)	416(22.7%)	130(22.1%)	150(24.3%)	136(21.8%)	0.511
Hx of Hip replacement	11(0.6%)	4(0.7%)	3(0.5%)	4(0.6%)	0.899
Hx of stroke	29(1.6%)	13(2.2%)	6(1.0%)	10(1.6%)	0.227
Hx of CHF	27(1.5%)	11(1.9%)	6(1.0%)	10(1.6%)	0.412
Hx of MI	**63(3.4%)**	**11(1.9%)**	**25(4.1%)**	**27(4.3%)**	**0.039**
Composite health issues**	537(29.4%)	165(28.1%)	189(30.7%)	183(29.3%)	0.607
FEV1/FVC ratio	78.36 ± 6.38	78.57 ± 6.09	78.40 ± 6.29	78.13 ± 6.74	0.505
Grip strength (kg-force)	24.0 ± 10.7	23.7 ± 10.6	24.5 ± 10.9	23.7 ± 10.5	0.345
Total metformin exposure (years)	**3.50 [0.00, 12.00]**	**0.00 [0.00, 4.50]**	**16.50 [8.38, 18.50]**	**0.50 [0.00, 6.50]**	***<*0.001**
Glycemic exposure (mean HbA1c, %)	**6.0 ± 0.7**	**6.0 ± 0.7**	**6.0 ± 0.7**	**6.1 ± 0.8**	***<*0.001**
Physical activity (Median MET-h/Wk)	10.88 [5.55, 18.83]	11.70 [6.42, 19.83]	10.77 [5.66, 18.51]	10.23 [4.94, 18.30]	0.379

Data shown are means ± SD or median [Q1, Q3] for continuous variables. *N* (col%) for categorical variables. *p*-values for ANOVA for group comparison for continuous variables and chi-squared for categorical variables. Bold font indicates a statistically significant association.

*Includes participants with neuropathy signs present based on monofilament test or symptoms from MNSI.

**Includes recent hospitalization (past 12 months), history of hip replacement, history of stroke, CHF or MI, and recent falls (past 12 months).

Because the interventions reduced the incidence of diabetes and its risk factors, diabetes, fasting glucose, and glycemic exposure differed between the randomization groups at the DPPOS Year 15, and were highest, on average, in the placebo group (Tables 1, 2). Diabetes had developed in 62.8% of lifestyle, 61.5% of metformin, and 68.5% of placebo participants. Other behaviors or health conditions potentially related to walking ability were not consistently or statistically significantly different across the DPP randomization groups. As a result of drug assignment in the randomized metformin group and use of metformin in all groups as a treatment for diabetes, the median metformin exposures by the DPPOS Year 15 were 0, 16.5, and 0.5 years in the lifestyle, metformin, and placebo groups, respectively.

**Table 2:** Variables associated with test completion and distance ≥200 m.

	Odds of test completion Complete: *N* = 1,697 Incomplete/Ineligible: N = 133				Odds of completion and distance ≥ 200 m Complete, distance ≥ 200 m: *N* = 1,530 Incomplete/Ineligible/Distance < 200 m: *N* = 300			
	Simple adjusted^1^		Fully adjusted^2^		Simple adjusted^1^		Fully adjusted^2^	
Characteristic (*N* = 1830)	aOR	95% CI	aOR	95% CI	aOR	95% CI	aOR	95% CI
DPP treatment assignment (Ref: Placebo)								
Lifestyle	1.40	0.89, 2.21	1.49	0.87, 2.59	1.18	0.86, 1.63	1.09	0.76, 1.56
Metformin	1.05	0.69, 1.59	0.86	0.53, 1.39	1.07	0.79, 1.45	0.94	0.66, 1.32
Baseline age group (Ref: 25–44 years)								
45–59 years	0.72	0.44, 1.14	0.72	0.41, 1.24	**0.61**	**0.44, 0.85**	**0.61**	**0.41, 0.89**
60+ years	**0.34**	**0.20, 0.59**	**0.30**	**0.14, 0.64**	**0.25**	**0.17, 0.38**	**0.28**	**0.17, 0.47**
Sex (Male v. Female)	0.88	0.60, 1.30	1.01	0.50, 2.05	1.05	0.79, 1.40	**0.58**	**0.35, 0.94**
Ethnicity (Ref: Non-Hispanic White)								
Non-Hispanic Black	0.69	0.44, 1.08	**0.58**	**0.34, 1.00**	**0.63**	**0.46, 0.88**	**0.58**	**0.40, 0.85**
Hispanic	1.04	0.63, 1.78	0.89	0.48, 1.71	**0.61**	**0.43, 0.87**	0.66	0.43, 1.00
American Indian	1.37	0.61, 3.67	1.44	0.53, 5.10	**1.99**	**1.02, 4.37**	**2.49**	**1.15, 6.25**
Asian	1.17	0.49, 3.44	0.49	0.19, 1.54	0.82	0.45, 1.57	0.71	0.36, 1.52
Education (Less than College vs. College)	**0.53**	**0.36, 0.78**	**0.55**	**0.35, 0.86**	**0.56**	**0.42, 0.75**	**0.58**	**0.43, 0.80**
Smoking (ref: Never)^3^								
Past	0.78	0.53, 1.15	0.67	0.43, 1.04	0.83	0.64, 1.10	0.75	0.55, 1.02
Current	0.40	0.18, 1.01	0.51	0.20, 1.63	0.60	0.31, 1.28	0.91	0.41, 2.28
BMI categories (ref: <30 kg/m^2^)^3^								
30–35 kg/m^2^	**0.59**	**0.36, 0.97**	0.56	0.30, 1.05	**0.70**	**0.50, 0.98**	0.86	0.57, 1.32
35–40 kg/m^2^	**0.45**	**0.26, 0.77**	0.60	0.26, 1.37	**0.53**	**0.36, 0.77**	0.82	0.48, 1.42
40+ kg/m^2^	**0.21**	**0.12, 0.37**	**0.31**	**0.11, 0.84**	**0.29**	**0.19, 0.45**	0.55	0.27, 1.12
Height (per 10 cm)^3^	0.97	0.73, 1.27	0.85	0.60, 1.19	**1.29**	**1.06, 1.58**	1.23	0.97, 1.55
Waist (per 15 cm)^4^	**0.56**	**0.45, 0.68**	0.79	0.55, 1.14	**0.64**	**0.55, 0.74**	0.80	0.62, 1.03
Grip strength (per 10 kg-force) ^3^	1.17	0.95, 1.48	1.16	0.91, 1.50	**1.51**	**1.27, 1.80**	**1.45**	**1.20, 1.76**
Median Physical Activity (per 5 MET-h/Wk)^5^	**1.11**	**1.03, 1.22**	1.03	0.94, 1.13	**1.13**	**1.06, 1.20**	**1.09**	**1.02, 1.17**
Neuropathy Signs/Symptoms (Present vs. Absent)^3^	**0.50**	**0.33, 0.74**	**0.59**	**0.37, 0.92**	**0.42**	**0.31, 0.55**	**0.45**	**0.33, 0.61**
Composite Health Issue (Present vs. Absent)^6^	**0.41**	**0.28, 0.59**	**0.46**	**0.30, 0.70**	**0.56**	**0.43, 0.73**	**0.64**	**0.48, 0.87**
Glycemic exposure (per 1% mean A1c)^7^	0.81	0.64, 1.04	0.98	0.74, 1.33	**0.76**	**0.64, 0.91**	0.91	0.74, 1.12

Bold font indicates a statistically significant association.

^1Odds ratio adjusted for age group, sex, ethnicity, and education.^

^2Odds ratios adjusted for all other variables in the table; adjusted for treatment group, sex, and ethnicity.^

^3^Collected at visit concurrent to 6MWT (year 15 visit).

^4^Collected 1 year prior to 6MWT (year 14 visit).

^5Median of self-reported physical activity measures from DPP baseline through DPPOS Year 12.^

^6^Includes recent hospitalization (past 12 months), history of hip replacement, history of stroke, CHF or MI, and recent falls (past 12 months).

^7Mean HbA1c of all available measures DPP baseline through DPPOS Year 15.^

Reasons for the 6MWT ineligibility included dizziness, shortness of breath, chest pain, unstable angina, high or low heart rate or blood pressure, or participant feeling unsafe to attempt the test. Table 2 shows variables associated with participants who completed the test and variables associated with a composite measure of ability to complete the test and walk a distance ≥200 m. A model adjusted for age group, sex, ethnicity, and education, and a model adjusted for all other variables in the table is presented (Table 2). Of the 1,830 participants, 133 did not complete the test (103 ineligible and 30 started but did not complete), and an additional 167 participants completed the test but walked <200 m. The randomization group was not associated with 6MWT completion or the ability to both complete and walk a distance ≥200 m. In contrast, older age, non-Hispanic Black ethnicity, lack of college education, higher BMI category, higher waist circumference, signs and symptoms of neuropathy, and presence of a health issue were each associated with a lower likelihood of test completion in fully-adjusted logistic regression models. These same factors were also associated with a lower likelihood of both completing the 6MWT and walking a distance ≥200 m in fully-adjusted logistic regression models; however, a higher likelihood of this outcome was associated with American Indian ethnicity, greater grip strength, and higher median physical activity.

The primary outcome of this analysis was the distance (m) walked in 6 min (6MWD). Table 3 presents the unadjusted mean distance walked by participant characteristics. There were no differences in distance walked by the randomization group but there were differences in all other variables presented. Neither distance walked nor gait speed was different for participants undergoing testing at sites with 10-m or 20-m courses.

**Table 3:** Mean distance walked (m) by participant characteristics at DPPOS Year 15 among participants who completed the 6MWT (N = 1,694).

Group	N	Mean	+/− SD	*p*-value*
**Overall**	1,694	361.5	105.7	
**Randomized group**				0.810
Lifestyle	552	361.5	107.8	
Metformin	568	363.5	105.4	
Placebo	574	359.5	104.2	
**Sex**				0.001
Female	1,172	355.4	100.8	
Male	522	375.1	115.0	
**Baseline age (years)**				<0.001
25–44 years	532	388.9	102.4	
45–59 years	944	363.1	102.7	
60+ years	218	287.6	91.5	
**Ethnicity**				<0.001
Non-Hispanic White	889	374.9	104.6	
Non-Hispanic Black	304	337.3	106.2	
Hispanic	286	332.6	106.1	
American Indian	128	382.2	79.4	
Asian	87	373.1	115.4	
**Education**				<0.001
College	1,293	372.0	104.7	
Less than College	401	327.5	101.9	
**Smoking**				0.02
Never	1,043	366.5	106.3	
Past	603	355.0	104.8	
Current	48	334.8	98.0	
**Diabetes**				0.02
No Diabetes	616	369.7	105.0	
Diabetes	1,077	356.9	105.9	
**BMI**				0.001
<30 kg/m^2^	722	374.7	113.2	
30–35 kg/m^2^	474	367.1	102.2	
35–40 kg/m^2^	295	346.1	93.0	
40+ kg/m^2^	202	324.2	92.1	
**Height**				<0.001
Low	566	339.6	108.0	
Medium	565	370.9	100.7	
High	562	374.3	104.9	
**Waist**				<0.001
Low	550	379.9	114.6	
Medium	547	370.7	102.2	
High	544	335.4	93.0	
**Mean HbA1c**				0.01
<5.5	312	371.1	106.6	
5.5–5.9	537	365.2	101.7	
6.0–6.4	342	356.4	108.0	
> = 6.5	327	344.7	103.3	
**Composite health issue**				<0.001
Not present	1,221	368.5	105.1	
Present	472	343.7	105.2	
**Neuropathy signs/Symptoms**				<0.001
Not present	880	380.5	98.2	
Present	762	339.9	110.0	
**Grip strength**				<0.001
Low	559	340.0	112.9	
Medium	558	363.5	101.7	
High	554	384.3	96.0	
**Physical activity**				<0.001
Low	550	339.4	101.8	
Medium	547	362.0	107.9	
High	544	385.2	101.5	

*p-value for unadjusted ANOVA type III F-test.*

Multivariable models for associations with distance walked among those who completed the test (*N* = 1,694) are shown in Table 4. In all three models, there was no significant association between the randomization group with 6MWD, with the greatest differences between randomization groups of approximately 4 m (or approximately 1% of the overall mean distance of 361.5 m). No interactions between the randomization groups with sex, ethnicity, and age on walking distance were observed.

**Table 4:** Association of randomized treatment assignment and selected covariates with distance walked (m).

Characteristic	Model 1			Model 2			Model 3		
	Beta	95% CI	*p*	Beta	95% CI	*p*	Beta	95% CI	*P*
*DPP treatment assignment (Ref: Placebo)*			0.6			0.9			>0.9
*Lifestyle*	3.7	−7.7, 15		−1.5	−12, 9.3		−2.5	−13, 8.3	
*Metformin*	6.2	−5.1, 17		1.4	−9.4, 12		−1.0	−12, 9.8	
Age (per 10 years)	−39	−44, −33	<0.001	−48	−54, −43	<0.001	−44	−50, −38	<0.001
Sex (Male vs. Female)	31	21, 42	<0.001	11	−2.9, 25	0.12	−9.8	−25, 5.2	0.2
Ethnicity (Ref: Non-Hispanic White)			<0.001			<0.001			<0.001
Non-Hispanic Black	−35	−47, −22		−33	−46, −21		−30	−43, −17	
Hispanic	−48	−61, −34		−41	−55, −27		−36	−50, −23	
American Indian	−2.0	−21, 17		5.3	−13, 24		5.2	−13, 24	
Asian	−14	−35, 8.0		−23	−45, −2.2		−19	−40, 2.8	
Education (Less than College vs. College)	−31	−42, −19	<0.001	−30	−40, −19	<0.001	−28	−38, −17	<0.001
Smoking (ref: Never) ^1^						0.001			0.007
Past				−7.4	−17, 2.2		−9.3	−19, 0.2	
Current				−47	−74, −20		−37	−63, −9.9	
BMI (per 5 kg/m^2^) ^1^				−13	−19, −6.2	<0.001	−14	−20, −7.6	<0.001
Height (per 10 cm) ^1^				19	11, 26	<0.001	15	8.0, 23	<0.001
Waist (per 15 cm) ^2^				−17	−25, −7.8	<0.001	−11	−20, −2.1	0.015
Grip strength (per 10 kg-force) ^1^							13	7.9, 18	<0.001
Habitual activity (per 5 MET-h) ^3^							3.4	1.6, 5.2	<0.001
Neuropathy signs/Symptoms (Present vs. Absent) ^1^							−19	−28, −9.4	<0.001
Composite health issue (Present vs. Absent) ^4^							−8.8	−19, 1.2	0.084
Glycemic exposure (per 1% mean A1c) ^5^							−9.6	−16, −3.0	0.004

Results of general linear models predicting total distance walked (meters) during the 6-min walk test among completers (*N* = 1,694). All models also adjusted for course length (10 m vs. 20 m): not significant. Note that interactions between Treatment*Sex, Treatment*Ethnicity, Treatment*Age were tested but none were statistically significant.

^1^Collected at visit concurrent to 6MWT (year 15 visit).

^2^Collected 1 year prior to 6MWT (year 14 visit).

^3Median of self-reported physical activity measures from DPP baseline through DPPOS Year 12.^

^4^Includes recent hospitalization (past 12 months), history of hip replacement, history of stroke, CHF or MI, and recent falls (past 12 months).

^5Mean HbA1c of all available measures DPP baseline through DPPOS Year 15.^

Supplementary Tables 2–6 present models evaluating associations of the 6MWD with diabetes, cumulative glycemic exposure, cumulative metformin exposure, BMI, and grip strength. There was no significant effect of concurrent diabetes after adjustment for adiposity, with further attenuation of the effect once adjusted for grip strength and physical activity. Cumulative glycemic exposure was significantly and inversely associated with 6MWD; each 1% increase in mean HbA1c was associated with a 9 m [95% CI -16, −2.5] lower walking distance. Cumulative metformin exposure had no significant association with the 6MWD. Concurrently measured BMI was inversely associated with the 6MWD with a 14 m [95% CI -20, −7.3] lower walking distance per 5 kg/m (2). Concurrently measured grip strength was positively associated with the 6MWD with a 13 m [95% CI 7.9, 18] greater walking distance per 10 kg force. In all models, age and education were inversely related to walking distance.

## Discussion

The distance walked in 6 min is a functional measure of mobility and walking endurance that decreases during the aging process, particularly in individuals with T2D. Both metformin and lifestyle interventions slow the progression of prediabetes to T2D, which may in turn impact walking endurance. In this study of DPPOS participants who were at risk of developing T2D, greater age and BMI negatively impacted walking endurance while physical activity and grip strength, a measure of muscle function, were positively associated with walking endurance. However, in this large cohort of participants followed longitudinally over 2 decades, there was no difference in walking endurance, as measured by the 6MWT, among those randomized to metformin, lifestyle, or placebo. Additionally, there was no association between cumulative metformin exposure and walking endurance as determined by the 6MWT.

We observed negative associations between 6MWT walking distance and age, BMI, health issues, and duration of diabetes, in line with previous studies (42). The Look AHEAD study assessed exercise capacity by a symptom-limited graded exercise treadmill test to voluntary exhaustion, in a large cohort of overweight/obese individuals with type 2 diabetes (43). The authors observed greater impairment of aerobic exercise capacity associated with greater levels of general and central obesity and a further reduction in fitness with increasing age and a longer duration of diabetes. In a study evaluating a healthy, younger cohort between 18 and 50 years of age, the 6MWD was not associated with age (44). However, in healthy older adults, older age has a negative association with distance walking during the 6MWT (21).

We also found an association between higher mean HbA1c and lower 6MWD suggesting that cumulative glycemic exposure negatively influences walking endurance. A study by Senefeld et al. did not find a difference in the 6MWD between T2D (HbA1c ≥7.0%) and controls; however, sample sizes were small (45, 46). The difference between these observations may lie in the study design and different glycemic exposures over time. Our study expands on this research and suggests that one possible effect of long-term hyperglycemia on 6MWT performance may be mediated via sustained hyperglycemia directly affecting skeletal muscle function and exercise adaptation (47). Finally, as with past research (48), we observed a positive association between walk distance and education, a marker of socioeconomic status.

In individuals with established T2D, lifestyle interventions that emphasize moderate-intensity physical activities have improved walking endurance. In the Look AHEAD study of adults with overweight/obesity and T2D, an intensive lifestyle intervention that included increasing physical activity had benefits in mobility, including an improvement in a 400-m walk test (49). Thus, while previous studies have shown a benefit of intensive lifestyle intervention on walking endurance in individuals with T2D during the active intervention period, treatment assignment did not show sustained benefits for walking endurance in DPP participants at risk for T2D during the DPPOS follow-up period, approximately 20 years after randomization.

The 6MWT is considered a useful and valid test of functional exercise capacity and endurance clinically in healthy aging populations (21, 50) and in those with chronic disease (51–54). Distance walking in 6 min may also represent an individual’s ability to perform certain activities of daily living since both require only a submaximal effort (19, 55, 56). In a previous analysis of DPPOS participants, those randomized to the lifestyle arm compared to the metformin or placebo arms had 37% lower odds of frailty (29). The components of the frailty assessment included walking speed (15-foot walk test), grip strength, physical activity, exhaustion, and unexplained weight loss (57). However, none of these individual frailty characteristics were different across the DPPOS randomization groups. Walking endurance, as measured by the 6MWT is not part of the frailty assessment (57). In the current analysis, we found that the 6MWD was associated with grip strength and habitual physical activity but we did not observe differences in walking distances by randomization group. Thus, while walking endurance and frailty are related (58), one possibility for our null findings may be that the effects of a lifestyle intervention wane over time when not maintained. The 6MWT assessment took place approximately 15 years after the end of the intensive lifestyle intervention phase of the DPP and only semi-annual lifestyle classes were provided for this group during DPPOS. However, the strong association between grip strength, physical activity, and 6MWT completion, as well as the 6MWD, suggests that maintaining an active lifestyle and maintaining muscle function after the end of the intervention may affect walking endurance in a population at risk for T2D. These findings are consistent with another small study evaluating an older cohort with T2D, where the 6MWD was positively associated with physical regular activity (48).

The strengths of our study include a long follow-up of a large, well-phenotyped, cohort of adults overweight and obese, all with a high risk of developing T2D at baseline, and continued assessment of glycemia semi-annually. Other strengths include a significant representation of Black, Hispanic, Native American, and Asian individuals, a high participation rate of those enrolled in DPPOS and still active (80%), and a comprehensive set of variables to examine the long-term effects of DPP interventions on walking endurance, and standardized procedures administered at diverse clinical sites in the United States.

There are a number of limitations to this analysis. The 6MWT was conducted approximately 15 years after the more active intervention period, and we cannot exclude the possibility that there may have been differences in the 6MWD among the groups had the test been performed earlier. Additionally, the 6MWT was not performed at randomization and was conducted only in a subset of the original DPP cohort (Supplementary Table 1). While we did not observe differences in attrition by treatment group, there is likely the presence of survivorship bias, where healthier individuals remain in the cohort. Furthermore, the conduct of the 6MWT was subject to variation by clinic, including differences in test environment and course length. Stop-watch time was recorded and exceeded 6 min for a subset of participants. Additional limitations include the potentially biased self-reports of leisure physical activity and the crossover in metformin use among the participants in the lifestyle and placebo cohorts who have been diagnosed with T2D. Metformin is a first-line treatment for type 2 diabetes; thus there is an increasing risk to the randomized analyses over time. However, total metformin exposure remained much higher in participants originally randomized to metformin. Similarly, the intensity of the lifestyle intervention was not sustained after the initial 3.2 years, and all participants were offered quarterly lifestyle classes during the DPPOS; however, participation in lifestyle classes was low across the intervention groups: approximately 80% of participants attended fewer than a quarter of the sessions offered, leading to reduced differences in weight between the groups. These features of the study may have reduced the relative effects of our interventions on 6MWT. Finally, while the 6MWT is a valid test of mobility and physical function, alternative measures such as the sit-to-stand, timed-up-and-go, or stair-climbing tests were not included in this analysis.

Although we did not observe a sustained effect of the originally randomized interventions on the 6MWT, we confirmed that the 6MWT is related to other measures of physical ability such as habitual physical activity and muscle strength (grip strength) in persons at risk for and with T2D. Our study also demonstrated that long-term metformin exposure does not negatively impact walking distance. While the randomized lifestyle treatment assignment was not associated with walking test distance, greater self-reported habitual physical activity, grip strength, and lower BMI were all associated with greater walking distances, suggesting potential long-term benefits of maintaining a healthy lifestyle. Given the importance of the 6MWT as a functional health measure reported in the literature, we anticipate that as DPPOS continues, it may predict future outcomes such as mortality and cognitive impairment.

## Author’s note

In accordance with the NIH Public Access Policy, we continue to provide all manuscripts to PubMed Central including this manuscript. DPP/DPPOS has provided the protocols and lifestyle and medication intervention manuals to the public through its public website (https://www.dppos.org). The DPPOS abides by the NIDDK data sharing policy and implementation guidance as required by the NIH/NIDDK (https://www.niddkrepository.org/studies/dppos/).

## Group membership of the Diabetes Prevention Program Research Group

Complete list of investigators in the Supplementary Appendix.

## Data Availability

The datasets presented in this study can be found in online repositories. The names of the repository/repositories and accession number(s) can be found at: https://www.niddkrepository.org/studies/dppos/.

## Author disclaimer

The content is solely the responsibility of the authors and does not necessarily represent the official views of the National Institutes of Health. The sponsor of this study was represented on the Steering Committee and played a part in study design, how the study was done, and publication. All authors in the writing group had access to all data. The opinions expressed are those of the study group and do not necessarily reflect the views of the funding agencies. A complete list of Centers, investigators, and staff can be found in the Appendix.
