# Supplementary material for: Long-term impact of Diabetes Prevention Program interventions on walking endurance
Source: Front Public Health. 2024 Dec 18;12:1470035. doi: 10.3389/fpubh.2024.1470035 (PMC11688401; doi:10.3389/fpubh.2024.1470035)
Supplement: Supplementary file 1 [file Supplementary_file_1.docx]

**Long-term Impact of Diabetes Prevention Program Interventions on Walking Endurance**

**Supplemental Materials**

**Supplemental Table 1. Among all randomized in DPP (N=3234), comparison of participants with a visit versus those without**

|  | All | | Without Visit | | With Visit | |  |
| --- | --- | --- | --- | --- | --- | --- | --- |
|  | **N** |  | **N** |  | **N** |  | **p-value** |
| Baseline Characteristics |  |  |  |  |  |  |  |
| Randomization Group |  |  |  |  |  |  | 0.2 |
| % Placebo |  | 1082 (33.5%) |  | 457 (32.5%) |  | 625 (34.2%) |  |
| % Metformin |  | 1073 (33.2%) |  | 456 (32.5%) |  | 617 (33.7%) |  |
| % ILS |  | 1079 (33.4%) |  | 491 (35.0%) |  | 588 (32.1%) |  |
| Age (years) | 3234 | 50.6 ± 10.7 | 1404 | 51.9 ± 12.2 | 1830 | 49.6 ± 9.2 | <.001 |
| Sex |  |  |  |  |  |  | 0.1 |
| % Male |  | 1043 (32.3%) |  | 474 (33.8%) |  | 569 (31.1%) |  |
| % Female |  | 2191 (67.7%) |  | 930 (66.2%) |  | 1261 (68.9%) |  |
| Race/ethnicity |  |  |  |  |  |  | <.001 |
| % NH White |  | 1768 (54.7%) |  | 811 (57.8%) |  | 957 (52.3%) |  |
| % NH Black |  | 645 (19.9%) |  | 307 (21.9%) |  | 338 (18.5%) |  |
| % Hispanic |  | 508 (15.7%) |  | 200 (14.2%) |  | 308 (16.8%) |  |
| % Am Indian |  | 171 (5.3%) |  | 37 (2.6%) |  | 134 (7.3%) |  |
| % Asian/Pac Isl |  | 142 (4.4%) |  | 49 (3.5%) |  | 93 (5.1%) |  |
| Education |  |  |  |  |  |  | 0.1 |
| % College + |  | 834 (25.8%) |  | 381 (27.1%) |  | 453 (24.8%) |  |
| % Less than college |  | 2400 (74.2%) |  | 1023 (72.9%) |  | 1377 (75.2%) |  |
| Weight (kg) | 3234 | 94.2 ± 20.3 |  | 96.4 ± 21.8 |  | 92.6 ± 18.9 |  |
| Height (cm) | 3234 | 166.4 ± 9.2 | 1404 | 166.9 ± 9.3 | 1830 | 166.1 ± 9.1 | 0.01 |
| BMI kg/m2 | 3234 | 34.0 ± 6.7 | 1404 | 34.6 ± 7.2 | 1830 | 33.5 ± 6.2 | <.001 |
| Waist (cm) | 3231 | 105.1 ± 14.5 | 1402 | 106.6 ± 15.2 | 1829 | 103.9 ± 13.9 | <.001 |
| Fasting Glucose (mg/dl) | 3234 | 106.5 ± 8.3 | 1404 | 107.2 ± 8.3 | 1830 | 106.0 ± 8.3 | <.001 |
| HbA1c | 3226 | 5.9 ± 0.5 | 1399 | 5.9 ± 0.5 | 1827 | 5.9 ± 0.5 | 0.2 |
| Status at end of Year 15* |  |  |  |  |  |  | <0.001 |
| Active |  | 2,120 (65.6%) |  | 300 (21.4%) |  | 1,820 (99.5%) |  |
| Deceased |  | 320 (9.9%) |  | 310 (22.1%) |  | 10 (0.5%) |  |
| Inactive |  | 333 (10.3%) |  | 333 (23.7%) |  | 0 (0.0%) |  |
| Never Enrolled in DPPOS |  | 338 (10.5%) |  | 338 (24.1%) |  | 0 (0.0%) |  |
| Withdrawn |  | 123 (3.8%) |  | 123 (8.8%) |  | 0 (0.0%) |  |

*Status is last study status at the close of the Year 15 visits (November 2017). A small group of participants completed year 15 visits and were subsequently deceased. For this table, those who never enrolled in DPPOS and those who refused enrollment are combined.

**Supplementary Table 2. – Association of *Year 15 diabetes* with distance walked (m).**

| **Characteristic** | **Model 1** | | | **Model 2** | | | **Model 3** | | |
| --- | --- | --- | --- | --- | --- | --- | --- | --- | --- |
|  | **Beta** | **95% CI** | **p** | **Beta** | **95% CI** | **p** | **Beta** | **95% CI** | **P** |
| *Diabetes (No Diabetes v Diabetes)^0^* | 16 | 6.1, 25 | 0.001 | 8.5 | -0.92, 18 | 0.077 | 6.0 | -3.4, 15 | 0.2 |
| DPP Treatment Assignment (Ref: Placebo) |  |  | 0.7 |  |  | 0.9 |  |  | >0.9 |
| Lifestyle | 2.6 | -8.8, 14 |  | -2.1 | -13, 8.7 |  | -1.9 | -13, 9.0 |  |
| Metformin | 4.9 | -6.4, 16 |  | 0.68 | -10, 12 |  | 0.1 | -11, 11 |  |
| Age (per 10 years) | -39 | -45, -34 | <0.001 | -49 | -55, -43 | <0.001 | -43 | -50, -37 | <0.001 |
| Sex (Male v. Female) | 32 | 22, 43 | <0.001 | 12 | -2.3, 25 | 0.10 | -9.8 | -25, 5.2 | 0.2 |
| Race/Ethnicity (Ref: Non-Hispanic White) |  |  | <0.001 |  |  | <0.001 |  |  | <0.001 |
| Non-Hispanic Black | -33 | -45, -20 |  | -32 | -44, -20 |  | -33 | -46, -21 |  |
| Hispanic | -47 | -61, -33 |  | -41 | -54, -27 |  | -39 | -52, -25 |  |
| American Indian | -1.0 | -20, 18 |  | 5.6 | -13, 24 |  | 2.1 | -16, 20 |  |
| Asian | -12 | -34, 9.3 |  | -22 | -44, -1.1 |  | -21 | -42, 0.4 |  |
| Education (Less than College v. College) | -30 | -41, -19 | <0.001 | -29 | -40, -18 | <0.001 | -28 | -39, -17 | <0.001 |
| Smoking (ref: Never) ^1^ |  |  |  |  |  | 0.002 |  |  | 0.006 |
| Past |  |  |  | -7.1 | -17, 2.5 |  | -9.3 | -19, 0.3 |  |
| Current |  |  |  | -46 | -73, -19 |  | -36 | -63, -9.5 |  |
| BMI (per 5 kg/m^2^) ^1^ |  |  |  | -13 | -19, -6.5 | <0.001 | -14 | -20, -7.2 | <0.001 |
| Height (per 10cm) ^1^ |  |  |  | 18 | 11, 26 | <0.001 | 15 | 7.7, 22 | <0.001 |
| Waist (per 15 cm) ^2^ |  |  |  | -16 | -24, -6.8 | <0.001 | -12 | -21, -3.1 | 0.008 |
| Grip strength (per 10kg-force) ^1^ |  |  |  |  |  |  | 13 | 8.2, 19 | <0.001 |
| Habitual activity (per 5 MET-hours/week) ^3^ |  |  |  |  |  |  | 3.4 | 1.5, 5.2 | <0.001 |
| Neuropathy Signs/Symptoms (Present v Absent) ^1^ |  |  |  |  |  |  | -20 | -29, -10 | <0.001 |
| Composite Health Issue (Present v Absent) ^4^ |  |  |  |  |  |  | -9.1 | -19, 0.87 | 0.074 |

Results of general linear models predicting total length/distance walked (meters) during the 6-minute walk test among completers. All models also adjusted for course length (10m v 20m): not significant.

^0^Diabetes as of the visit concurrent to 6MWT (year 15 visit)

^1^Collected at visit concurrent to 6MWT (year 15 visit)

^2^Collected one year prior to 6MWT (year 14 visit)

^3^Median of self-reported physical activity measures from DPP baseline through DPPOS Year 12

^4^Includes recent hospitalization (past 12 months), history of hip replacement, history of stroke, CHF or MI and recent falls (past 12 months)

**Supplementary Table 3. – Association of Cumulative Glycemic Exposure (Mean HbA1c) with distance walked (m).**

| **Characteristic** | **Model 1** | | | **Model 2** | | | **Model 3** | | |
| --- | --- | --- | --- | --- | --- | --- | --- | --- | --- |
|  | **Beta** | **95% CI** | **p** | **Beta** | **95% CI** | **p** | **Beta** | **95% CI** | **P** |
| *Mean HbA1c (per 1% mean A1c)* | -16 | -23, -9.5 | <0.001 | -12 | -19, -5.9 | <0.001 | -9.6 | -16, -3.0 | 0.004 |
| DPP Treatment Assignment (Ref: Placebo) |  |  | 0.8 |  |  | 0.9 |  |  | >0.9 |
| Lifestyle | 2.0 | -9.3, 13 |  | -2.8 | -14, 8.0 |  | -2.5 | -13, 8.3 |  |
| Metformin | 3.5 | -7.8, 15 |  | -0.60 | -11, 10 |  | -1.0 | -12, 9.8 |  |
| Age (per 10 years) | -40 | -46, -35 | <0.001 | -50 | -55, -44 | <0.001 | -44 | -50, -38 | <0.001 |
| Sex (Male v. Female) | 33 | 22, 43 | <0.001 | 11 | -2.6, 25 | 0.11 | -9.8 | -25, 5.2 | 0.2 |
| Race/Ethnicity (Ref: Non-Hispanic White) |  |  | <0.001 |  |  | <0.001 |  |  | <0.001 |
| Non-Hispanic Black | -28 | -41, -15 |  | -28 | -41, -15 |  | -30 | -43, -17 |  |
| Hispanic | -44 | -58, -30 |  | -38 | -52, -24 |  | -36 | -50, -23 |  |
| American Indian | 4.3 | -14, 23 |  | 9.6 | -8.7, 28 |  | 5.2 | -13, 24 |  |
| Asian | -10 | -32, 11 |  | -20 | -41, 1.7 |  | -19 | -40, 2.8 |  |
| Education (Less than College v. College) | -30 | -41, -18 | <0.001 | -29 | -39, -18 | <0.001 | -28 | -38, -17 | <0.001 |
| Smoking (ref: Never) ^1^ |  |  |  |  |  | 0.002 |  |  | 0.007 |
| Past |  |  |  | -7.2 | -17, 2.3 |  | -9.3 | -19, 0.2 |  |
| Current |  |  |  | -46 | -73, -20 |  | -37 | -63, -9.9 |  |
| BMI (per 5 kg/m^2^) ^1^ |  |  |  | -13 | -20, -7.0 | <0.001 | -14 | -20, -7.6 | <0.001 |
| Height (per 10cm) ^1^ |  |  |  | 19 | 11, 26 | <0.001 | 15 | 8.0, 23 | <0.001 |
| Waist (per 15 cm) ^2^ |  |  |  | -14 | -23, -5.5 | 0.001 | -11 | -20, -2.1 | 0.015 |
| Grip strength (per 10kg-force) ^1^ |  |  |  |  |  |  | 13 | 7.9, 18 | <0.001 |
| Habitual activity (per 5 MET-hours/week) ^3^ |  |  |  |  |  |  | 3.4 | 1.6, 5.2 | <0.001 |
| Neuropathy Signs/Symptoms (Present v Absent) ^1^ |  |  |  |  |  |  | -19 | -28, -9.4 | <0.001 |
| Composite Health Issue (Present v Absent) ^4^ |  |  |  |  |  |  | -8.8 | -19, 1.2 | 0.084 |

Results of general linear models predicting total distance walked (meters) during the 6-minute walk test among completers. All models also adjusted for course length (10m v 20m): not significant.

^1^Collected at visit concurrent to 6MWT (year 15 visit)

^2^Collected one year prior to 6MWT (year 14 visit)

^3^Median of self-reported physical activity measures from DPP baseline through DPPOS Year 12

^4^Includes recent hospitalization (past 12 months), history of hip replacement, history of stroke, CHF or MI and recent falls (past 12 months)

**Supplementary Table 4. – Association of *cumulative metformin exposure (years)* with distance walked (m).**

| **Characteristic** | **Model 1** | | | **Model 2** | | | **Model 3** | | |
| --- | --- | --- | --- | --- | --- | --- | --- | --- | --- |
|  | **Beta** | **95% CI** | **p** | **Beta** | **95% CI** | **p** | **Beta** | **95% CI** | **P** |
| *Metformin exposure (years)^0^* | -0.81 | -4.1, 2.5 | 0.6 | -0.3 | -3.5, 2.8 | 0.8 | 0.3 | -2.8, 3.4 | 0.9 |
| Age (per 10 years) | -39 | -44, -33 | <0.001 | -48 | -54, -43 | <0.001 | -44 | -50, -38 | <0.001 |
| Sex (Male v. Female) | 32 | 21, 42 | <0.001 | 11 | -2.8, 25 | 0.12 | -9.7 | -25, 5.2 | 0.2 |
| Race/Ethnicity (Ref: Non-Hispanic White) |  |  | <0.001 |  |  | <0.001 |  |  | <0.001 |
| Non-Hispanic Black | -34 | -47, -22 |  | -33 | -46, -21 |  | -30 | -43, -17 |  |
| Hispanic | -48 | -61, -34 |  | -41 | -55, -28 |  | -37 | -50, -23 |  |
| American Indian | -2.0 | -21, 17 |  | 5.2 | -13, 23 |  | 5.1 | -13, 23 |  |
| Asian | -14 | -36, 7.5 |  | -24 | -45, -2.8 |  | -19 | -40, 2.7 |  |
| Education (Less than College v. College) | -31 | -42, -20 | <0.001 | -30 | -40, -19 | <0.001 | -28 | -38, -17 | <0.001 |
| Smoking (ref: Never) ^1^ |  |  |  |  |  | 0.001 |  |  | 0.007 |
| Past |  |  |  | -7.5 | -17, 2.1 |  | -9.3 | -19, 0.2 |  |
| Current |  |  |  | -47 | -74, -21 |  | -36 | -63, -9.8 |  |
| BMI (per 5 kg/m^2^) ^1^ |  |  |  | -13 | -19, -6.2 | <0.001 | -14 | -20, -7.5 | <0.001 |
| Height (per 10cm) ^1^ |  |  |  | 19 | 11, 26 | <0.001 | 15 | 8.0, 23 | <0.001 |
| Waist (per 15 cm) ^2^ |  |  |  | -17 | -25, -7.7 | <0.001 | -11 | -20, -2.1 | 0.015 |
| Grip strength (per 10kg-force) ^1^ |  |  |  |  |  |  | 13 | 7.9, 18 | <0.001 |
| Habitual activity (per 5 MET-hours/week) ^3^ |  |  |  |  |  |  | 3.4 | 1.5, 5.2 | <0.001 |
| Neuropathy Signs/Symptoms (Present v Absent) ^1^ |  |  |  |  |  |  | -19 | -28, -9.4 | <0.001 |
| Composite Health Issue (Present v Absent) ^4^ |  |  |  |  |  |  | -8.8 | -19, 1.2 | 0.085 |
| Glycemic exposure (per 1% mean A1c) ^5^ |  |  |  |  |  |  | -9.6 | -16, -3.0 | 0.005 |

Results of general linear models predicting total length/distance walked (meters) during the 6-minute walk test among completers. All models also adjusted for course length (10m v 20m): not significant.

^0^Cumulative exposure to study and non-study metformin, years of exposure through visit concurrent to 6MWT

^1^Collected at visit concurrent to 6MWT (year 15 visit)

^2^Collected one year prior to 6MWT (year 14 visit)

^3^Median of self-reported physical activity measures from DPP baseline through DPPOS Year 12

^4^Includes recent hospitalization (past 12 months), history of hip replacement, history of stroke, CHF or MI and recent falls (past 12 months)

^5^Mean HbA1c of all available measures DPP baseline through DPPOS Year 15

**Supplementary Table 5. – Association of *Year 15 Body Mass Index (per 5 kg/m^2^)* with distance walked (m).**

| **Characteristic** | **Model 1** | | | **Model 2** | | | **Model 3** | | |
| --- | --- | --- | --- | --- | --- | --- | --- | --- | --- |
|  | **Beta** | **95% CI** | **p** | **Beta** | **95% CI** | **p** | **Beta** | **95% CI** | **P** |
| *BMI (per 5 kg/m^2^)* ^1^ | -23 | -26, -19 | <0.001 | -13 | -19, -6.2 | <0.001 | -14 | -20, -7.6 | <0.001 |
| DPP Treatment Assignment (Ref: Placebo) |  |  | 0.8 |  |  | 0.9 |  |  | >0.9 |
| Lifestyle | 1.6 | -9.3, 13 |  | -1.5 | -12, 9.3 |  | -2.5 | -13, 8.3 |  |
| Metformin | 3.2 | -7.6, 14 |  | 1.4 | -9.4, 12 |  | -1.0 | -12, 9.8 |  |
| Age (per 10 years) | -51 | -56, -45 | <0.001 | -48 | -54, -43 | <0.001 | -44 | -50, -38 | <0.001 |
| Sex (Male v. Female) | 26 | 16, 36 | <0.001 | 11 | -2.9, 25 | 0.12 | -9.8 | -25, 5.2 | 0.2 |
| Race/Ethnicity (Ref: Non-Hispanic White) |  |  | <0.001 |  |  | <0.001 |  |  | <0.001 |
| Non-Hispanic Black | -31 | -43, -19 |  | -33 | -46, -21 |  | -30 | -43, -17 |  |
| Hispanic | -49 | -62, -36 |  | -41 | -55, -27 |  | -36 | -50, -23 |  |
| American Indian | -6.4 | -24, 11 |  | 5.3 | -13, 24 |  | 5.2 | -13, 24 |  |
| Asian | -30 | -51, -9.6 |  | -23 | -45, -2.2 |  | -19 | -40, 2.8 |  |
| Education (Less than College v. College) | -30 | -41, -19 | <0.001 | -30 | -40, -19 | <0.001 | -28 | -38, -17 | <0.001 |
| Smoking (ref: Never) ^1^ |  |  |  |  |  | 0.001 |  |  | 0.007 |
| Past |  |  |  | -7.4 | -17, 2.2 |  | -9.3 | -19, 0.2 |  |
| Current |  |  |  | -47 | -74, -20 |  | -37 | -63, -9.9 |  |
| Height (per 10cm) ^1^ |  |  |  | 19 | 11, 26 | <0.001 | 15 | 8.0, 23 | <0.001 |
| Waist (per 15 cm) ^2^ |  |  |  | -17 | -25, -7.8 | <0.001 | -11 | -20, -2.1 | 0.015 |
| Grip strength (per 10kg-force) ^1^ |  |  |  |  |  |  | 13 | 7.9, 18 | <0.001 |
| Habitual activity (per 5 MET-hours/week) ^3^ |  |  |  |  |  |  | 3.4 | 1.6, 5.2 | <0.001 |
| Neuropathy Signs/Symptoms (Present v Absent) ^1^ |  |  |  |  |  |  | -19 | -28, -9.4 | <0.001 |
| Composite Health Issue (Present v Absent) ^4^ |  |  |  |  |  |  | -8.8 | -19, 1.2 | 0.084 |
| Glycemic exposure (per 1% mean A1c) ^5^ |  |  |  |  |  |  | -9.6 | -16, -3.0 | 0.004 |

Results of general linear models predicting total length/distance walked (meters) during the 6-minute walk test among completers. All models also adjusted for course length (10m v 20m): not significant.

^1^Collected at visit concurrent to 6MWT (year 15 visit)

^2^Collected one year prior to 6MWT (year 14 visit)

^3^Median of self-reported physical activity measures from DPP baseline through DPPOS Year 12

^4^Includes recent hospitalization (past 12 months), history of hip replacement, history of stroke, CHF or MI and recent falls (past 12 months)

^5^Mean HbA1c of all available measures DPP baseline through DPPOS Year 15

**Supplementary Table 6. – Association of *Year 15 Grip Strength* with distance walked (m).**

| **Characteristic** | **Model 1** | | | **Model 2** | | | **Model 3** | | |
| --- | --- | --- | --- | --- | --- | --- | --- | --- | --- |
|  | **Beta** | **95% CI** | **p** | **Beta** | **95% CI** | **p** | **Beta** | **95% CI** | **P** |
| *Grip strength (per 10kg-force)* ^1^ | 16 | 11, 21 | <0.001 | 14 | 8.5, 19 | <0.001 | 13 | 7.9, 18 | <0.001 |
| DPP Treatment Assignment (Ref: Placebo) |  |  | 0.8 |  |  | 0.9 |  |  | >0.9 |
| Lifestyle | 3.5 | -7.8, 15 |  | -0.90 | -12, 9.9 |  | -2.5 | -13, 8.3 |  |
| Metformin | 6.1 | -5.1, 17 |  | 1.7 | -9.0, 12 |  | -1.0 | -12, 9.8 |  |
| Age (per 10 years) | -35 | -40, -29 | <0.001 | -46 | -51, -40 | <0.001 | -44 | -50, -38 | <0.001 |
| Sex (Male v. Female) | 11 | -1.7, 23 | 0.090 | -1.9 | -17, 13 | 0.8 | -9.8 | -25, 5.2 | 0.2 |
| Race/Ethnicity (Ref: Non-Hispanic White) |  |  | <0.001 |  |  | <0.001 |  |  | <0.001 |
| Non-Hispanic Black | -37 | -50, -25 |  | -35 | -47, -23 |  | -30 | -43, -17 |  |
| Hispanic | -45 | -59, -31 |  | -42 | -55, -28 |  | -36 | -50, -23 |  |
| American Indian | 1.3 | -17, 20 |  | 6.1 | -12, 24 |  | 5.2 | -13, 24 |  |
| Asian | -9.0 | -31, 13 |  | -21 | -42, 0.2 |  | -19 | -40, 2.8 |  |
| Education (Less than College v. College) | -30 | -41, -19 | <0.001 | -29 | -40, -18 | <0.001 | -28 | -38, -17 | <0.001 |
| Smoking (ref: Never) ^1^ |  |  |  |  |  | 0.004 |  |  | 0.007 |
| Past |  |  |  | -7.0 | -17, 2.5 |  | -9.3 | -19, 0.2 |  |
| Current |  |  |  | -42 | -69, -15 |  | -37 | -63, -9.9 |  |
| BMI (per 5 kg/m^2^) ^1^ |  |  |  | -13 | -20, -6.9 | <0.001 | -14 | -20, -7.6 | <0.001 |
| Height (per 10cm) ^1^ |  |  |  | 15 | 7.1, 22 | <0.001 | 15 | 8.0, 23 | <0.001 |
| Waist (per 15 cm) ^2^ |  |  |  | -16 | -25, -7.1 | <0.001 | -11 | -20, -2.1 | 0.015 |
| Habitual activity (per 5 MET-hours/week) ^3^ |  |  |  |  |  |  | 3.4 | 1.6, 5.2 | <0.001 |
| Neuropathy Signs/Symptoms (Present v Absent) ^1^ |  |  |  |  |  |  | -19 | -28, -9.4 | <0.001 |
| Composite Health Issue (Present v Absent) ^4^ |  |  |  |  |  |  | -8.8 | -19, 1.2 | 0.084 |
| Glycemic exposure (per 1% mean A1c) ^5^ |  |  |  |  |  |  | -9.6 | -16, -3.0 | 0.004 |

Results of general linear models predicting total length/distance walked (meters) during the 6-minute walk test among completers. All models also adjusted for course length (10m v 20m): not significant.

^1^Collected at visit concurrent to 6MWT (year 15 visit)

^2^Collected one year prior to 6MWT (year 14 visit)

^3^Median of self-reported physical activity measures from DPP baseline through DPPOS Year 12

^4^Includes recent hospitalization (past 12 months), history of hip replacement, history of stroke, CHF or MI and recent falls (past 12 months)

^5^Mean HbA1c of all available measures DPP baseline through DPPOS Year 15

**Supplemental Figure 1. Inclusion Flow**
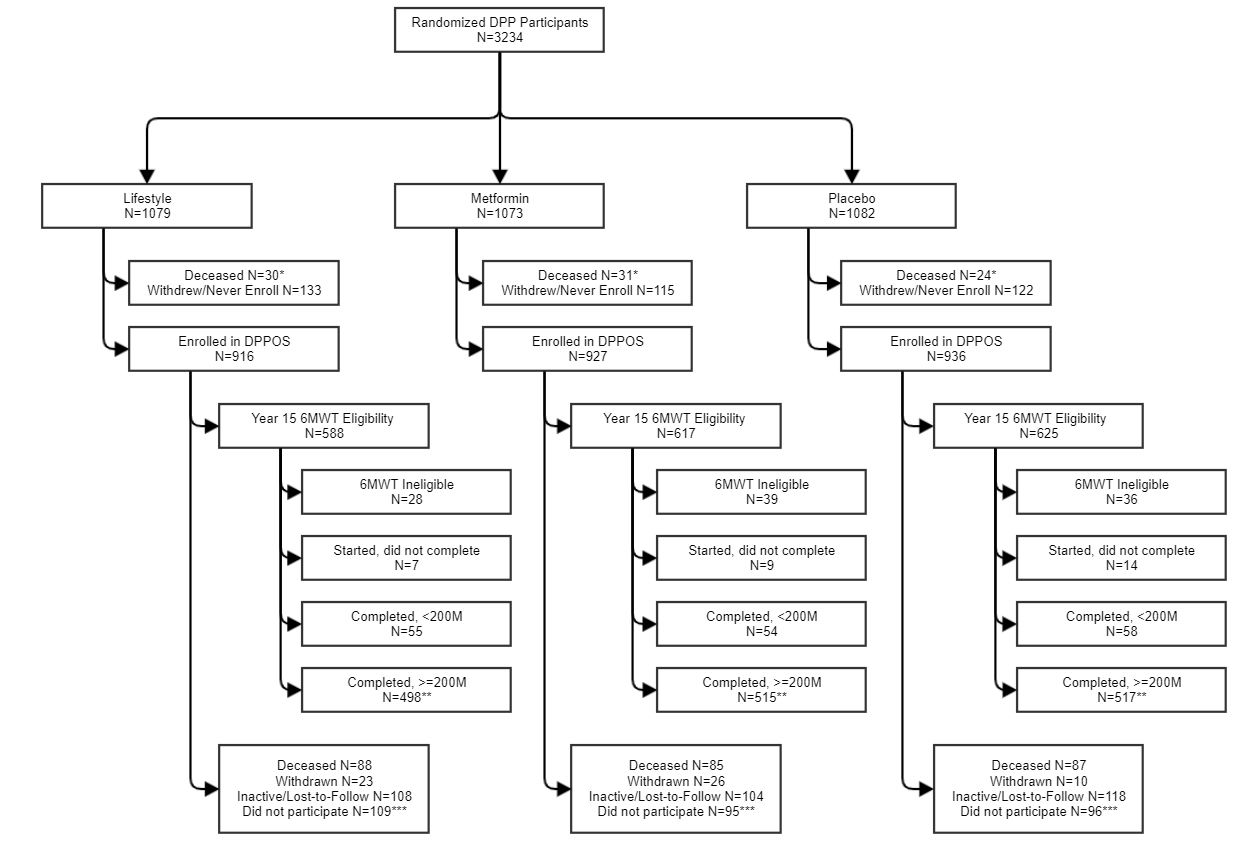


*Enrollment into DPPOS remained open throughout DPPOS thus “deceased” includes participants who did not enroll and died at any time.

**Participants with stopwatch time ≥7 minutes were excluded from subsequent analyses with total distance walked as the outcome (one participant per treatment group).

***This includes participants at a clinic that was unable to participate along with participants who completed their visits by phone or outside of the clinic (e.g. at home).
