# Supplementary material for: Long-term impact of Diabetes Prevention Program interventions on walking endurance
Source: Front Public Health. 2024 Dec 18;12:1470035. doi: 10.3389/fpubh.2024.1470035 (PMC11688401; doi:10.3389/fpubh.2024.1470035)
Supplement: Supplementary file 2 [file Supplementary_file_2.docx]

**Pennington Biomedical Research Center**

**(Baton Rouge, LA)**

George A. Bray, MD*

Kishore M. Gadde, MD*

Iris W. Culbert, BSN, RN, CCRC**

Jennifer Arceneaux RN, BSN**

Annie Chatellier, RN, CCRC**

Amber Dragg RD, LDN**

Catherine M. Champagne, PhD, RD

Crystal Duncan, LPN

Barbara Eberhardt, RD, LDN

Frank Greenway, MD

Fonda G. Guillory, LPN

April A. Herbert, RD

Michael L. Jeffirs, LPN

Betty M. Kennedy, MPA

Erma Levy, RD

Monica Lockett, LPN

Jennifer C. Lovejoy, PhD

Laura H. Morris, BS

Lee E. Melancon, BA, BS

Donna H. Ryan, MD

Deborah A. Sanford, LPN

Kenneth G. Smith, BS, MT

Lisa L. Smith, BS

Julia A. St.Amant, RTR

Richard T. Tulley, PhD

Paula C. Vicknair, MS, RD

Donald Williamson, PhD

Jeffery J. Zachwieja, PhD

**University of Chicago (Chicago, IL)**

Kenneth S. Polonsky, MD*

Janet Tobian, MD, PhD*

David A. Ehrmann, MD*

Margaret J. Matulik, RN, BSN**

Karla A. Temple, PhD, RDN, LDN**

Bart Clark, MD

Kirsten Czech, MS

Catherine DeSandre, BA

Brittnie Dotson, MS

Ruthanne Hilbrich, RD

Wylie McNabb, EdD

Ann R. Semenske, MS, RD

Celeste C. Thomas, MD

**Jefferson Medical College (Philadelphia, PA)**

Jose F. Caro, MD*

Kevin Furlong, DO*

Barry J. Goldstein, MD, PhD*

Pamela G. Watson, RN, ScD*

Kellie A. Smith, RN, MSN**

Jewel Mendoza, RN, BSN**

Marsha Simmons, CCRP**

Wendi Wildman, RN**

Renee Liberoni, MPH

John Spandorfer, MD

Constance Pepe, MS, RD

**University of Miami (Miami, FL)**

Richard P. Donahue, PhD*

Ronald B. Goldberg, MD*

Ronald Prineas, MD, PhD*

Jeanette Calles, MSEd**

Anna Giannella, RD, MS**

Patricia Rowe, MPA**

Juliet Sanguily, RN**

Paul Cassanova-Romero, MD

Sumaya Castillo-Florez, MPH

Hermes J. Florez, MD

Rajesh Garg, MD

Lascelles Kirby, MS

Olga Lara

Carmen Larreal

Valerie McLymont, RN

Jadell Mendez

Arlette Perry, PhD

Patrice Saab, PhD

Bertha Veciana

**The University of Texas Health Science Center**

**(San Antonio, TX)**

Steven M. Haffner, MD, MPH*

Helen P. Hazuda, PhD*

Maria G. Montez, RN, MSHP, CDE**

Kathy Hattaway, RD, MS

Juan Isaac, RN, BSN**

Carlos Lorenzo, MD, PhD

Arlene Martinez, RN, BSN, CDE

Monica Salazar

Tatiana Walker, RD, MS, CDE

**University of Colorado (Denver, CO)**

Dana Dabelea, MD, PhD*

Richard F. Hamman, MD, DrPH*

Patricia V. Nash, MS**

Sheila C. Steinke, MS**

Lisa Testaverde, MS**

Jennifer Truong, MPH**

Denise R. Anderson, RN, BSN

Larry B. Ballonoff, MD

Alexis Bouffard, MA, RN, BSN

Brian Bucca OD, FAOO

B. Ned Calonge, MD, MPH

Lynne Delve

Martha Farago, RN

James O. Hill, PhD

Shelley R. Hoyer, BS

Tonya Jenkins, RD, CDE

Bonnie T. Jortberg, MS, RD, CDE

Dione Lenz, RN, BSN, CDE

Marsha Miller, MS, RD

Thomas Nilan, BS

Leigh Perreault, MD

David W. Price, MD

Judith G. Regensteiner, PhD

Emily B. Schroeder, MD

Helen Seagle, MS, RD

Carissa M. Smith, BS

Brent VanDorsten, PhD

**Joslin Diabetes Center (Boston, MA)**

Edward S. Horton, MD*

Medha Munshi, MD*

Kathleen E. Lawton, RN**

Sharon D. Jackson,CCRC,MS, RD, CDE**

Catherine S. Poirier, RN, BSN**

Kati Swift, RN, BSN**

Ronald A. Arky, MD

Marybeth Bryant

Jacqueline P. Burke, BSN

Enrique Caballero, MD

Karen M. Callaphan, BA

Barbara Fargnoli, RD

Therese Franklin

Om P. Ganda, MD

Ashley Guidi, BS

Mathew Guido, BA

Alan M. Jacobsen, MD

Lyn M. Kula, RD

Margaret Kocal, RN, CDE

Lori Lambert, MS, RD, LD

Kathleen E. Lawton, RN

Sarah Ledbury, Med, RD

Maureen A. Malloy, BS

Roeland J.W. Middelbeek, MD

Maryanne Nicosia, MS, RD

Cathryn F. Oldmixon, RN

Jocelyn Pan, BS, MPH

Marizel Quitingon

Riley Rainville, BS

Stacy Rubtchinsky, BS

Ellen W. Seely, MD

Jessica Sansoucy, BS

Dana Schweizer, BSN

Donald Simonson, MD

Fannie Smith, MD

Caren G. Solomon, MD, MPH

Jeanne Spellman, RD

James Warram, MD

**VA Puget Sound Health Care System and University of Washington (Seattle, WA)**

Steven E. Kahn, MB, ChB*

Brenda K. Montgomery, RN, BSN, CDE**

Basma Fattaleh, BA **

Celeste Colegrove, BS

Wilfred Fujimoto, MD

Robert H. Knopp, MD

Edward W. Lipkin, MD

Michelle Marr, BA

Ivy Morgan-Taggart

Anne Murillo, BS

Kayla O’Neal, BS

Dace Trence, MD

Lonnese Taylor, RN, BS

April Thomas, RD, MPH, CDE

Elaine C. Tsai, MD, MPH

**University of Tennessee (Memphis, TN)**

Samuel Dagogo-Jack, MD, DSc, FRCP, FACP*

Abbas E. Kitabchi, PhD, MD, FACP*

Mary E. Murphy, RN, MS, CDE, MBA**

Laura Taylor, RN, BSN, CDE**

Jennifer Dolgoff, RN, BSN**

William B. Applegate, MD, MPH

Michael Bryer-Ash, MD

Debra Clark, LPN

Sandra L. Frieson, RN

Uzoma Ibebuogu, MD

Raed Imseis, MD

Helen Lambeth, RN, BSN

Lynne C. Lichtermann, RN, BSN

Hooman Oktaei, MD

Harriet Ricks

Lily M.K. Rutledge, RN, BSN

Amy R. Sherman, RD, LD

Clara M. Smith, RD, MHP, LDN

Judith E. Soberman, MD

Beverly Williams-Cleaves, MD

Avnisha Patel, MLT

Ebenezer A. Nyenwe, MD, FACP

Ethel Faye Hampton, R.N.

**Northwestern University’s Feinberg School of Medicine (Chicago, IL)**

Boyd E. Metzger, MD*

Mark E. Molitch, MD*

Amisha Wallia, MD*

Mariana K. Johnson, MS, RN**

Daphne T. Adelman, MBA, RN

Catherine Behrends

Michelle Cook, MS

Marian Fitzgibbon, PhD

Mimi M. Giles, MS, RD

Deloris Heard, MA

Cheryl K.H. Johnson, MS, RN

Diane Larsen, BS

Anne Lowe, BS

Megan Lyman, BS

David McPherson, MD

Samsam C. Penn, BA

Thomas Pitts, MD

Renee Reinhart, RN, MS

Susan Roston, RN, RD

Pamela A. Schinleber, RN, MS

Matthew O’Brien, MD

Monica Hartmuller, MS, RN

**Massachusetts General Hospital (Boston, MA)**

David M. Nathan, MD*

Charles McKitrick, BSN**

Heather Turgeon, BSN**

Mary Larkin, MSN, RN, CDCES**

Marielle Mugford, BA**

Kathy Abbott

Ellen Anderson, MS, RD

Laurie Bissett, MS, RD

Kristy Bondi, BS

Enrico Cagliero, MD

Jose C. Florez, MD, PhD+

Linda Delahanty, MS, RD

Valerie Goldman, MS, RD

Elaine Grassa

Lindsery Gurry BSN, RN, CDCES

Kali D’Anna

Fernelle Leandre BS

Peter Lou, MD

Alexandra Poulos

Elyse Raymond, BS

Valerie Ripley, BS

Christine Stevens, RN

Beverly Tseng

Kathy Chu, BA

Nopporn Thangthaeng, PhD, BSN, CDCES**

**University of California-San Diego (La Jolla, CA)**

Jerrold M. Olefsky, MD*

Elizabeth Barrett-Connor, MD*

Sunder Mudaliar, MD*

Maria Rosario Araneta, PhD*

Mary Lou Carrion-Petersen, RN, BSN**

Karen Vejvoda, RN, BSN, CDE, CCRC**

Sarah Bassiouni, MPH

Madeline Beltran, RN, BSN, CDE

Lauren N. Claravall, BS

Jonalle M. Dowden, BS

Steven V. Edelman, MD

Pranav Garimella, MBBS

Robert R. Henry, MD

Javiva Horne, RD

Marycie Lamkin, RN

Simona Szerdi Janesch, BA

Diana Leos

William Polonsky, PhD

Rosa Ruiz

Jean Smith, RN

Jennifer Torio-Hurley

**Columbia University (New York, NY)**

F. Xavier Pi-Sunyer, MD*

Blandine Laferrere, MD, PhD*

Jane E. Lee, MS**

Susan Hagamen, MS, RN, CDE**

David B. Allison, PhD

Nnenna Agharanya

Nancy J. Aronoff, MS, RD

Maria Baldo

Jill P. Crandall, MD

Sandra T. Foo, MD

Kim Kelly-Dinham

Jose A. Luchsinger, MD, MPH

Carmen Pal, MD

Kathy Parkes, RN

Mary Beth Pena, RN

Ellen S. Rooney, BA

Gretchen E.H. Van Wye, MA

Kristine A. Viscovich, ANP

**Indiana University (Indianapolis, IN)**

Mary de Groot, PhD*

David G. Marrero, PhD*

Kieren J. Mather, MD*

Melvin J. Prince, MD*

Susie M. Kelly, RN, CDE**

Marcia A. Jackson**

Gina McAtee**

Paula Putenney, RN**

Ronald T. Ackermann, MD

Carolyn M. Cantrell

Yolanda F. Dotson, BS

Edwin S. Fineberg, MD

Megan Fultz

John C. Guare, PhD

Angela Hadden

James M. Ignaut, MA

Marion S. Kirkman, MD

Erin O’Kelly Phillips

Kisha L Pinner

Beverly D. Porter, MSN

Paris J. Roach, MD

Nancy D. Rowland, BS, MS

Madelyn L. Wheeler, RD

**Medstar Research Institute (Washington, DC)**

Vanita Aroda, MD*

Michelle Magee, MD*

Robert E. Ratner, MD*

Michelle Magee, MD*

Gretchen Youssef, RD, CDE**

Sue Shapiro, RN, BSN, CCRC**

Natalie Andon, RN

Catherine Bavido-Arrage, MS, RD, LD

Geraldine Boggs, MSN, RN

Marjorie Bronsord, MS, RD, CDE

Ernestine Brown

Holly Love Burkott, RN

Wayman W. Cheatham, MD

Susan Cola

Cindy Evans

Peggy Gibbs

Tracy Kellum, MS, RD, CDE

Lilia Leon

Milvia Lagarda

Claresa Levatan, MD

Milajurine Lindsay

Asha K. Nair, BS

Jean Park, MD

Maureen Passaro, MD

Angela Silverman

Gabriel Uwaifo, MD

Debra Wells-Thayer, NP, CDE

Renee Wiggins, RD

**University of Southern California/UCLA Research Center (Alhambra, CA)**

Mohammed F. Saad, MD*

Karol Watson, MD*

Christine Darwin, MD

Preethi Srikanthan, MD

Tamara Horwich, MD

Adrian Casillas, MD

Arleen Brown, MD

Maria Budget**

Sujata Jinagouda, MD**

Medhat Botrous, MD**

Anthony Sosa**

Sameh Tadros**

Khan Akbar, MD

Claudia Conzues

Perpetua Magpuri

Carmen Muro

Noemi Neira

Kathy Ngo

Michelle Chan

Veronica Villarreal

Amer Rassam, MD

Debra Waters

Kathy Xapthalamous

**Washington University (St. Louis, MO)**

Julio V. Santiago, MD*

Samuel Dagogo-Jack, MD, MSc, FRCP, FACP*

Neil H. White, MD, CDE*

Angela L. Brown, MD*

Samia Das, MS, MBA, RD, LD**

Prajakta Khare-Ranade, MSc, RDN, LD**

Tamara Stich, RN, MSN, CDE**

Ana Santiago, RN

Edwin Fisher, PhD

Emma Hurt, RN

Tracy Jones, RN

Michelle Kerr, RD

Lucy Ryder, RN

Cormarie Wernimont, RD, LD

**Johns Hopkins School of Medicine**

**(Baltimore, MD)**

Sherita Hill Golden, MD, MHS, FAHA*

Christopher D. Saudek, MD*

Vanessa Bradley, BA**

Emily Sullivan, MEd, RN**

Tracy Whittington, BS**

Caroline Abbas

Adrienne Allen

Frederick L. Brancati, MD, MHS

Sharon Cappelli

Jeanne M. Clark, MD

Jeanne B. Charleston, RN, MSN

Janice Freel

Katherine Horak, RD

Alicia Greene

Dawn Jiggetts

Deloris Johnson

Hope Joseph

Kimberly Loman

Nestoras Mathioudakis, MD, MHS

Henry Mosley

John Reusing

Richard R. Rubin, PhD

Alafia Samuels, MD

Thomas Shields

Shawne Stephens

Kerry J. Stewart, EdD

LeeLana Thomas

Evonne Utsey

Paula Williamson

**University of New Mexico (Albuquerque, NM)**

David S. Schade, MD*

Karwyn S. Adams, RN, MSN**

Janene L. Canady, RN, CDE**

Carolyn Johannes, RN, CDE**

Claire Hemphill, RN, BSN**

Penny Hyde, RN, BSN**

Leslie F. Atler, PhD

Patrick J. Boyle, MD

Mark R. Burge, MD

Lisa Chai, RN

Kathleen Colleran, MD

Ateka Fondino

Ysela Gonzales

Doris A. Hernandez-McGinnis

Patricia Katz, LPN

Carolyn King, Med

Julia Middendorf, RN

Amer Rassam, MD

Sofya Rubinchik, MD

Willette Senter, RD

Debra Waters, PhD

**Albert Einstein College of Medicine (Bronx, NY)**

Jill Crandall, MD*

Harry Shamoon, MD*

Janet O. Brown, RN, MPH, MSN**

Gilda Trandafirescu, MD**

Danielle Powell, MPH**

Norica Tomuta, MD

Elsie Adorno, BS

Liane Cox, MS, RD

Helena Duffy, MS, C-ANP

Samuel Engel, MD

Allison Friedler, BS

Angela Goldstein, FNP-C, NPP, CSW

Crystal J. Howard-Century, MA

Jennifer Lukin, BA

Stacey Kloiber, RN

Nadege Longchamp, LPN

Helen Martinez, RN, MSN, FNP-C

Dorothy Pompi, BA

Jonathan Scheindlin, MD

Elissa Violino, RD, MS

Elizabeth A. Walker PhD, RN

Judith Wylie-Rosett, EdD, RD

Elise Zimmerman, RD, MS

Joel Zonszein, MD

**University of Pittsburgh (Pittsburgh, PA)**

Trevor Orchard, MD*

Elizabeth Venditti, PhD*

Rena R. Wing, PhD*

Susan Jeffries, RN, MSN**

Gaye Koenning, MS, RD**

M. Kaye Kramer, BSN, MPH**

Marie Smith, RN, BSN**

Susan Barr, BS

Catherine Benchoff

Miriam Boraz, PhD

Lisa Clifford, BS

Rebecca Culyba, BS

Marlene Frazier

Ryan Gilligan, BS

Stephanie Guimond, BS

Susan Harrier, MLT

Louann Harris, RN

Andrea Kriska, PhD

Qurashia Manjoo, MD

Monica Mullen, MHP, RD

Alicia Noel, BS

Amy Otto, PhD

Jessica Pettigrew, CMA

Bonny Rockette-Wagner, PhD

Debra Rubinstein, MD

Linda Semler, MS, RD

Cheryl F. Smith, PhD

Valarie Weinzierl, MPH

Katherine V. Williams, MD, MPH

Tara Wilson, BA

Bonnie Gillis, MS, RD, LDN

**University of Hawaii (Honolulu, HI)**

Marjorie K. Mau, MD*

Narleen K. Baker-Ladao, BS**

John S. Melish, MD

Richard F. Arakaki, MD*

Renee W. Latimer, BSN, MPH**

Mae K. Isonaga, RD, MPH**

Ralph Beddow, MD

Nina E. Bermudez, MS

Lorna Dias, AA

Jillian Inouye, RN, PhD

Kathy Mikami, BS, RD

Pharis Mohideen, MD

Sharon K. Odom, RD, MPH

Raynette U. Perry, AA

Robin E. Yamamoto, CDE, RD

**Southwest American Indian Centers**

**(Phoenix, AZ; Shiprock, NM; Zuni, NM)**

William C. Knowler, MD, DrPH*+

Robert L. Hanson, MD, MPH*

Harelda Anderson, LMSW**

Norman Cooeyate**

Charlotte Dodge**

Mary A. Hoskin, RD, MS**

Carol A. Percy, RN, MS**

Alvera Enote**

Camille Natewa**

Kelly J. Acton, MD, MPH

Vickie L. Andre, RN, FNP

Rosalyn Barber

Shandiin Begay, MPH

Peter H. Bennett, MB, FRCP

Mary Beth Benson, RN, BSN

Evelyn C. Bird, RD, MPH

Brenda A. Broussard, RD, MPH, MBA, CDE

Brian C. Bucca, OD, FAAO

Marcella Chavez, RN, AS

Sherron Cook

Jeff Curtis, MD

Tara Dacawyma

Matthew S. Doughty, MD

Roberta Duncan, RD

Cyndy Edgerton, RD

Jacqueline M. Ghahate

Justin Glass, MD

Martia Glass, MD

Dorothy Gohdes, MD

Wendy Grant, MD

Ellie Horse

Louise E. Ingraham, MS, RD, LN

Merry Jackson

Priscilla Jay

Roylen S. Kaskalla

Karen Kavena, ANP

David Kessler, MD

Kathleen M. Kobus, RNC-ANP

Jonathan Krakoff, MD

Jason Kurland, MD

Catherine Manus, LPN

Cherie McCabe

Sara Michaels, MD

Tina Morgan

Yolanda Nashboo

Julie A. Nelson, RD

Steven Poirier, MD

Evette Polczynski, MD

Christopher Piromalli, DO

Mike Reidy, MD

Jeanine Roumain, MD, MPH

Debra Rowse, MD

Robert J. Roy

Sandra Sangster, RD

Janet Sewenemewa

Miranda Smart

Chelsea Spencer

Darryl Tonemah, PhD

Rachel Williams, FNP

Charlton Wilson, MD

Michelle Yazzie

**George Washington University Biostatistics Center (DPP Coordinating Center Rockville, MD)**

Raymond Bain, PhD*

Sarah Fowler, PhD*

Marinella Temprosa, PhD*

Michael D. Larsen, PhD*

Kathleen Jablonski, PhD*

Tina Brenneman**

Sharon L. Edelstein, ScM**

Solome Abebe, MS

Julie Bamdad, MS

Melanie Barkalow

Joel Bethepu

Tsedenia Bezabeh

Anna Bowers

Nicole Butler

Jackie Callaghan

Caitlin E. Carter

Costas Christophi, PhD

Gregory M. Dwyer, MPH

Mary Foulkes, PhD

Yuping Gao

Robert Gooding

Adrienne Gottlieb

Kristina L. Grimes

Nisha Grover-Fairchild, MPH

Lori Haffner, MS

Heather Hoffman, PhD

Steve Jones

Tara L. Jones

Richard Katz, MD

Preethy Kolinjivadi, MS

John M. Lachin, ScD

Yong Ma, PhD

Pamela Mucik

Robert Orlosky

Qing Pan, PhD

Susan Reamer

James Rochon, PhD

Alla Sapozhnikova

Hanna Sherif, MS

Charlotte Stimpson

Ashley Hogan Tjaden, MPH

Fredricka Walker-Murray

Audrey McMaster

Rhea Mundra

Hannah Rapoport

Nolan Kuenster

**Lifestyle Resource Core**

Elizabeth M. Venditti, PhD*

Andrea M. Kriska, PhD

Linda Semler, MS, RD, LDN

Valerie Weinzierl, MPH

**Central Biochemistry Laboratory (Seattle, WA)**

Santica Marcovina, PhD, ScD*

F. Alan Aldrich**

Jessica Harting**

John Albers, PhD

Greg Strylewicz, PhD

**Central Biochemistry Laboratory (Minneapolis, MN)**

Robert Janicek, MT, CLS*

Anthony Killeen, MD, PhD

Deanna Gabrielson, MLS (ASCP)^CM^, PMP

**NIH/NIDDK (Bethesda, MD)**

R. Eastman, MD

Judith Fradkin, MD

Sanford Garfield, PhD

Christine Lee, MD, MS

**Centers for Disease Control & Prevention**

**(Atlanta, GA)**

Edward Gregg, PhD

Ping Zhang, PhD

**Carotid Ultrasound**

Dan O’Leary, MD*

Gregory Evans

**Coronary Artery Calcification Reading Center**

Matthew Budoff, MD

Chris Dailing

**CT Scan Reading Center**

Elizabeth Stamm, MD*

**Dual Energy X-ray Absorptiometry Reading Center (San Francisco, CA)**

Ann Schwartz, PhD

Caroline Navy

Lisa Palermo, MS

**Epidemiological Cardiology Research Center- Epicare (Winston-Salem, NC)**

Pentti Rautaharju, MD, PhD*

Ronald J. Prineas, MD, PhD**

Teresa Alexander

Charles Campbell, MS

Sharon Hall

Yabing Li, MD

Margaret Mills

Nancy Pemberton, MS

Farida Rautaharju, PhD

Zhuming Zhang, MD

Elsayed Z. Soliman, MD*

Julie Hu, MSc

Susan Hensley, BS

Lisa Keasler

Tonya Taylor

**Fundus Photo Reading Center (Madison, WI)**

Barbara Blodi, MD*

Ronald Danis, MD*

Matthew Davis, MD*

Larry Hubbard*

Ryan Endres**

Deborah Elsas**

Samantha Johnson**

Dawn Myers**

Nancy Barrett

Heather Baumhauer

Wendy Benz

Holly Cohn

Ellie Corkery

Kristi Dohm

Amitha Domalpally, MD, PhD

Vonnie Gama

Anne Goulding

Andy Ewen

Cynthia Hurtenbach

Daniel Lawrence

Kyle McDaniel

Jeong Pak

James Reimers

Ruth Shaw

Maria Swift

Pamela Vargo, CRA

Sheila Watson

**Neurocognitive Assessment Group**

Jose A. Luchsinger, MD, MPH

Jennifer Manly, PhD

**Nutrition Coding Center (Columbia, SC)**

Elizabeth Mayer-Davis, PhD*

Robert R. Moran, PhD**

**Quality of Well-Being Center (La Jolla, CA)**

Ted Ganiats, MD*

Kristin David, MHP*

Andrew J. Sarkin, PhD*

Erik Groessl, PhD

Naomi Katzir

Helen Chong, MA

**University of Michigan (Ann Arbor, MI)**

William H. Herman, MD, MPH

Michael Brändle, MD, MS

Morton B. Brown, PhD

**+Genetics Working Group**

Jose C. Florez, MD, PhD^1, 2^

David Altshuler, MD, PhD^1, 2^

Liana K. Billings, MD^1^

Ling Chen, MS^1^

Maegan Harden, BS^2^

Robert L. Hanson, MD, MPH^3^

William C. Knowler, MD, DrPH^3^

Toni I. Pollin, PhD^4^

Alan R. Shuldiner, MD^4^

Kathleen Jablonski, PhD^5^

Paul W. Franks, PhD, MPhil, MS^6, 7, 8^

Marie-France Hivert, MD^8^

1=Massachusetts General Hospital

2=Broad Institute

3=NIDDK

4=University of Maryland

5=Coordinating Center

6=Lund University, Sweden

7=Umeå University, Sweden

8=Harvard School of Public Health
